# Looking beyond Body Structure and Function: ICF Foci and Who Is Being Assessed in Research about Adolescents and Young Adults with Cerebral Palsy—A Scoping Review

**DOI:** 10.3390/ijerph21060670

**Published:** 2024-05-24

**Authors:** Camila Araújo Santos Santana, Peter Rosenbaum, Jet van der Kemp, Ana Carolina de Campos

**Affiliations:** 1 Physiotherapy Department, Child Development Analysis Laboratory (LADI), Federal University of São Carlos (UFSCar), São Carlos 13565-905, SP, Brazil; accampos@ufscar.br; 2 CanChild Centre for Childhood Disability Research, McMaster University, Hamilton, ON L8S 4L8, Canada; 3 Center of Excellence for Rehabilitation Medicine, UMC Utrecht Brain Center, University Medical Center Utrecht and De Hoogstraat Rehabilitation, 3584 Utrecht, The Netherlands

**Keywords:** cerebral palsy, health development, adolescents, young adult, transition to adult life, research focus

## Abstract

Purpose: The purpose of this study is to summarize the ICF foci, looking beyond body structures and function, and to analyze who has been assessed in research about adolescents and young adults (AYAs) with CP in the phase of transition to adulthood. Method: Medline, EMBASE, PsycINFO, and CINAHL databases were searched using terms related to cerebral palsy, adolescents/young adults, health development, participation, and independence. Studies including youth with CP (13–30 years old) published in English from 2014 to 2021 were considered. The methods of assessment reported in the included studies were used to identify the ICF foci and who was assessed. Results: In this study, 86 studies were reviewed. The main ICF foci are activity and participation (51% of the studies), personal factors (23%), ICF not covered (14%), ICF not defined (9%), with environmental factors being the least focused ICF component (3%). Most studies assessed AYAs directly (49% of studies). Conclusions: Activity- and participation-related constructs are the leading research focus of studies, and more attention is needed concerning environmental factors. AYAs are the main source of information, and the perspectives of other key figures are also being valued. To bridge the gap between child and adult health care, a broader view of health development and approaches to explore AYA developmental issues must be taken.

## 1. Introduction

In 2001, the World Health Organization published the International Classification of Functioning, Disability and Health (ICF) with a framework for health that highlights a network of components that influence the functional status of all individuals. Moving beyond the impairments in body structure and function (BSF) traditionally addressed under a biomedical standpoint, components of activity and participation (AP), personal factors (PFs), and environmental factors (EFs) have achieved recognition under this holistic view of health [1]. Additionally, the Life Course Health Development concepts (LCHD) proposed by Halfon et al. (2014) [2] expand our perspective on health development, stating that health is a result of continuous interactions between the person and their environment. On-time experiences for this life stage are important for well-being, as well as for ensuring that the person’s voice is heard, thus grounding the development of the necessary supports and opportunities throughout their life course.

These biopsychosocial concepts encourage us to stop thinking of disabilities as just a biological or medical phenomenon ‘within the person’ and encourage us to think also about the impact of the impairments on a person’s functioning and life experience in general [2,3]. In this sense, over the years, the delivery of healthcare has evolved from a paternalistic, provider-driven, and disease-focused approach towards one of person-centered care, which puts the client in the center, engages them in decision making, develops their knowledge, and fosters self-care behavior [4,5,6].

Looking to the key developmental stages of life, research focusing on understanding the transition to adulthood of adolescents and young adults with chronic child-onset health conditions such as cerebral palsy (CP) [7] shows that the inadequate or limited healthcare services offered during childhood and adolescence may have a negative impact later on the lives of adults with CP [8], or that they at least fail to promote optimal health in the next stages of life.

Quality of life (QoL) is one health indicator that provides a broad picture of a person’s perception of life in several aspects [9] during their development. The childhood QoL level has been reported as a consistent predictor of QoL in adolescence [10]. For young adults with CP and more severe impairments, despite the increase in the QoL in social relationships, psychological well-being can decrease over the years [11], which can impact their health development in adult life.

With this in mind, supporting the development of other demands and capabilities related to adulthood, such as self-confidence, maintaining relationships, having a job, and independent living, are important aspects to be addressed in health care [12,13]; however, these elements are often a neglected aspect of the care delivered to young people with CP in the phase of transitioning to adulthood [14].

Research that has provided a voice to youths with CP reveals that they need more support to receive and seek information regarding transitioning to adulthood and overcoming environmental barriers, and they highlighted the desire for more strategies to promote real-life experiences related to adult life through their development [15,16]. Youth with CP also reinforce that participation is a key component of healthy living, and skills such as the self-management of their own health and self-advocacy should also be part of the care delivered to them; whereas skills such as fine and gross motor functions may have less importance, except if these functions directly impact their ability to participate [17].

However, impairments in BSF frequently are the main, if not the only, focus of interventions [18,19] aiming to improve the overall health [3] of individuals with CP. A literature review about interventions for children with CP outlined the lack of approaches focusing on aspects beyond BSF [20], which may lead clinicians to miss the opportunity to include other key aspects to improve the health of people with CP in their plan of care. To change this, instead of primarily targeting the achievement of abilities for functional skills, service providers are being challenged to expand their focus and promote rehabilitation strategies to improve the daily living capacities that are important to the person [17].

Under this approach, there is less emphasis on promoting typical development or ‘normal’ patterns, and more focus on finding solutions to enable AYAs to be autonomous and functional in activities which are meaningful to them, even in the face of the significant limitations of BSF, and even if they do things differently from typical peers [3].

Thus, healthcare professionals are encouraged to consider all of the ICF components as equally important entry points to promote the health of people with disabilities [21]. In this scenario, a broad exploration of the recent literature can contribute to identifying research patterns that need to change for the knowledge in this field to be expanded and support practice. Thus, we aimed to conduct a scoping review to summarize the ICF foci, to look beyond body structures and function, and to analyze who has been assessed in research about adolescents and young adults (AYAs) with CP in the phase of transitioning to adulthood. We specifically targeted research exploring health development, independence, and participation. Results from this review can indicate research gaps and guide future research and clinical practice on matters that need to be further addressed, thus contributing to improvements in the health development of young people with CP during the transition to adult life.

## 2. Materials and Methods

This review is registered at OSF Registries and can be found at https://doi.org/10.17605/OSF.IO/J7XWZ (accessed on 14 May 2024). As a literature review, this scoping review does not require ethical committee approval. This review summarizes evidence published from 2014 to 2021, the last update being performed in February 2022. All the methodological steps for scoping reviews proposed by Arksey and O’Malley (2005) [22] were followed. The PRISMA checklist for scoping reviews [23] is presented in Supplementary Information S1.

### 2.1. Identifying the Research Question

To present our research question in a structured manner, we used the ECLIPSE framework proposed by Wildridge and Bell (2002) [24]. Different from the PICO framework, which could be more useful for reviews focusing on clinical practice questions, the ECLIPSE framework (E: expectations, C: client group, L: location, I: impact, P: professionals, SE: service to deliver information) aims to assist searches for health planning and information management. Thus, our research question was as follows: Based on the published literature (P) between 2014 and 2021 (L), and looking beyond ICF body structure and function (E), which issues are emphasized and who was assessed (I) regarding the global health development, participation, or independence (SE) of adolescents and young adults with cerebral palsy (C)?

### 2.2. Identifying Relevant Studies

Considering that, during adolescence and young adulthood, youth are transitioning to adult life and developing globally as unique persons, the following five key areas of interest were defined for the literature search based on our focus of interest and the general outcomes expected in adult life: cerebral palsy, adolescents/young adults, health development, participation, and independence. Librarians with experience in health sciences were consulted to guide the structure of the search strategy and provide useful databases to be searched. The search strategy defined was adapted according to each database, and a complete example can be seen in Supplementary Information S2. Through the OVID interface, we searched Medline, EMBASE, and PsycINFO, and, through the EBSCOhost, we searched CINAHL, limiting the articles to those published between 2014 and 2021 in the English language.

### 2.3. Study Selection

The Covidence systematic review software [25] was used to manage the review process. In applying the inclusion and exclusion criteria, two independent reviewers (CASS and ACC or JVDK) screened all of the titles and abstracts. A third reviewer analyzed the disagreements (PR or ACC), and the first author (ACC) reviewed the full text and extracted data from the included papers. The criteria established were as follows in Table 1:

### 2.4. Charting the Data

From the included studies, we extracted the first author name, publication year, first author country, title, the study aims, study type according to the authors’ description, participant characteristics (study groups, age, the CP participants’ Gross Motor Function Classification System (GMFCS) [26], Manual Ability Classification System (MACS) [27], Communication Function Classification System (CFCS) [28], Eating and Drinking Ability Classification System (EDACS) [29], and Visual Function Classification System (VFCS) [30] levels), the assessment method, and who was assessed as described in the study’s methodology.

### 2.5. Collating, Summarizing, and Reporting the Results

Based on the methods of assessment used in each study (e.g., the scale or other method used to measure the study’s main outcome of interest) and its constructs, we linked the findings to the most appropriate ICF domain in order to understand the research focus. For this, we followed the ICF linking rules proposed by Cieza et al. (2002; 2019) [31,32]. The ICF universe can encompass various constructs assessed by most tools or assessment methods; however, the ICF components and categories cannot be broad or specific enough to cover all of the possible constructs being assessed in the studies. Thus, some constructs needed to be linked as ‘ICF—not covered’ (ICF-NC) when the meaning of the construct is not covered by any ICF component (e.g., quality of life) [32]. In cases where the construct’s meaning does not clearly belong to any ICF category, it could be linked as ‘ICF—not defined’ (ICF-ND) [32].

In addition, during the ICF linking process, we consulted the List of Pediatric Assessment Tools Categorized by ICF Model proposed by the American Physical Therapy Association [33], and, for tools that were not on this list, we consulted the Toolbox proposed by Schiariti et al. (2017) [34], the Rehabilitation Measures Database [35], or we searched constructs of the variables of interest using the ICF browser on the WHO ICF website [36].

The first author completed the first linking of all assessment methods, another author (ACC) independently checked the categorization, possible disagreements were solved via a consensus meeting (CASS and ACC), and all authors reviewed the final linking. To analyze who was assessed in the studies, we considered from who the studie’s variables were collected from, considering who completed the questionnaires or other outcomes of interest according to each author’s description in the methods.

## 3. Results

**Figure 1:**
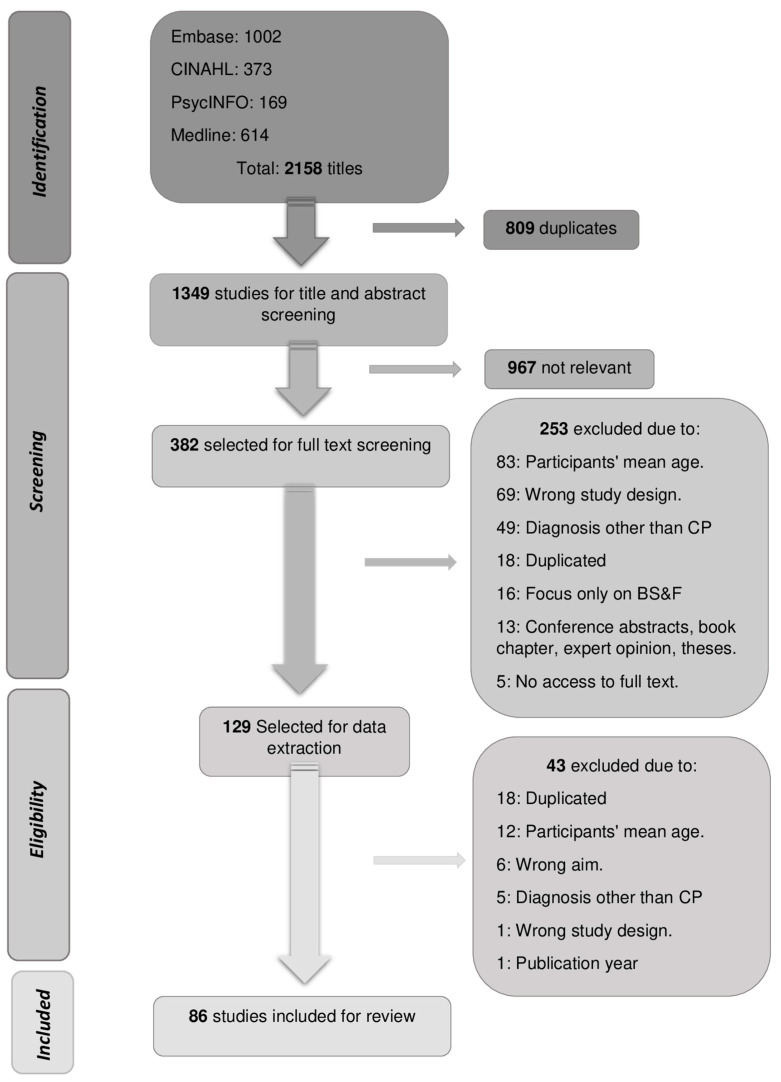
PRISMA Flowchart with the study selection process.

Studies published between January 2014 and December 2021 were retrieved from the databases, with the last search being updated in February 2022. After the study selection, 86 studies were included in this review for data summarization. The complete selection process can be seen in the PRISMA flowchart in Figure 1.

### 3.1. Studies Characteristics

Within the time reviewed, an average of 10 studies (SD ± 3.6) were published each year addressing the general health development, independence, or participation of young people with CP between 13 and 30 years of age. Despite including studies from 22 countries, high-income countries were the main source of publications in this field, of which the most frequently included were the United States of America (N= 17 studies), the Netherlands (N = 15), Canada (N = 13), Australia (n = 8), and the United Kingdom (N = 6). Among low- and middle-income countries, Brazil and South Africa were the most frequently represented, with three studies each.

Excluding the review studies, which comprise a diverse number of participants, our scoping review summarizes data from approximately 52,500 individuals, of whom around 43,570 have CP. Only the study by Liljenquist et al. (2018) [37] collected data from 35,290 individuals with CP. Furthermore, 42 studies focused on adolescents (with the mean age between 13–17 years) and 33 studies focused on young adults (mean age between 18–30 years), while 14 studies did not report the participants’ mean age, only stating that the age range was between 13 and 30 years.

The majority of studies did not have a comparison or control group (N = 60), focusing only on AYAs with CP. There was not a specific focus on any CP functional profile, with a relatively balanced representation of all manual, gross motor, and communication functional levels. The GMFCS was the classification system most frequently used (N = 63), followed by the MACS (N = 17) and CFCS (N = 8); only seven studies used all three of these common classification systems. No study used all five classification systems (GMFCS, MACS, CFCS, EDACS, VFCS) to characterize their population, and eighteen studies did not use any.

A wide range of methodologies was used across the studies, including observational, integrative knowledge translation, retrospective, randomized controlled trials, and others. Qualitative studies (N = 27) and longitudinal or cross-sectional studies (N = 15 each) were the most frequent methods used. In qualitative studies, interviews with open-ended questions and focus groups were the most common assessment method used. Supplementary Information S3 summarizes the included studies’ characteristics.

### 3.2. ICF Foci beyond BSF

Most studies did not specifically mention or organize their methods of assessment according to the ICF components. When categorizing the outcome measures based on the ICF components, among the eighty-six included studies, only five studies [38,39,40,41,42] included assessments comprising all other ICF components (AP, PFs, and EFs), and twenty-eight included more than one of these ICF components, thus assessing activity- and/or participation-related outcomes in combination with PFs and/or EFs in the same study.

Among the ICF components, the main focus of studies was related to AP (N = 31), with twenty studies assessing both AP, and six studies focusing mainly on activity-related constructs, such as functional classifications, mobility performance, and performance in activities of daily life. Participation-related outcomes were the main focus of five of the thirty-one studies. The Life-H was the most used tool when assessing participation.

The second ICF component most frequently focused on was PFs, with 24 studies choosing to assess only constructs related to this, such as AYAs’ age, gender, educational level, and perspectives about different issues, such as having a disability, socialization, and employment. The personal perspectives of AYAs and other persons, such as family members and health care providers, were explored, commonly using open-ended questions. The most frequently used tool to assess PFs was the Strengths and Difficulties Questionnaire (SDQ), usually answered by the parents, to screen the youth’s behaviour.When interviewing the AYAs, one of the tools used was the General Self-Efficacy Scale (GSEE), aiming to explore the AYAs’ perceptions of self-efficacy.

The ICF component that was focused on the least were EFs, with only three studies [43,44,45] solely focusing on it and exploring issues related to healthcare services, such as care needs for AYAs transitioning to adulthood, and aspects related to parental distress. Together with other ICF components, studies explored other EFs, such as socioeconomic status, living situation, and aid needs. The assessment tools varied in this section, and the Craig Hospital Inventory of Environmental Factors (CHIEF), Pediatric Rehabilitation Intervention Measure of Engagement—Service Provider version (PRIME-SP), and the Measure of Processes of Care-20 (MPOC-20) were some of the tools used.

Excepting AP, PFs, and EFs, some studies explored constructs not covered (ICF-NC) or not defined (ICF-ND) among these main ICF domains. Thus, following the ICF linking rule used in this review, the outcomes of assessment in 15 studies were categorized as ICF-NC, as all of them assessed aspects related to quality of life (QoL). KIDSCREEN was the most used tool to assess QoL, chosen by six studies, followed by the SF-36, which was used in four studies.

**Figure 2:**
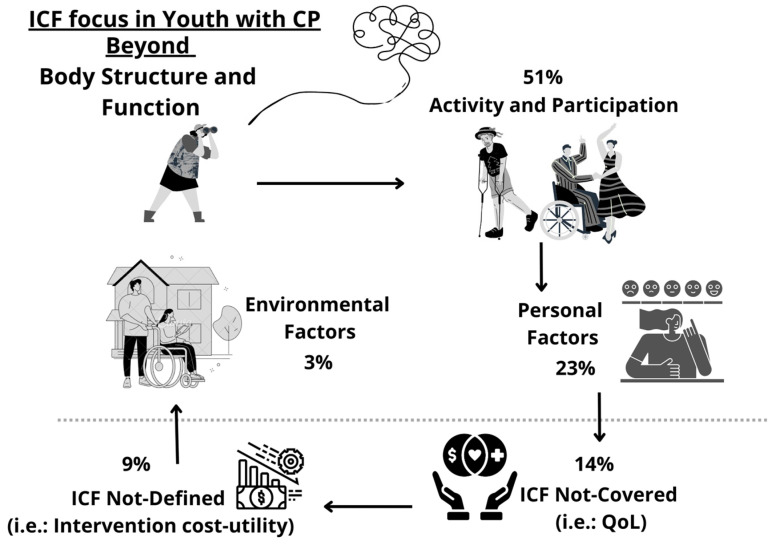
ICF foci of studies addressing aspects related to health development, independence, or the participation of AYAs with CP. Legend: ICF: International Classification of Functioning, Disability and Health; CP: Cerebral Palsy; QoL: Quality of Life.

Nine studies assessed constructs classified as ICF-ND, such as intervention cost-utility or summaries of database information. Looking beyond BSF, the distribution of the ICF foci of studies explored in this review is represented in Figure 2.

Despite not being the aim of this review, BSF is an important and frequent target in studies. Thus, we also took note of the methods used to assess these categories in the included studies. Among the 86 included studies, 59 did not assess any constructs related to BSF along with their primary outcomes. Only two studies assessed both components of body structure and body function along with AP, PFs, and/or EFs [46,47]. Specifically, body function was assessed in 25 studies, with constructs related to pain, fatigue, and cognition. No studies assessed only body structures together with other ICF components. These details are also included in Supplementary Information S3.

### 3.3. Who Was Assessed

**Figure 3:**
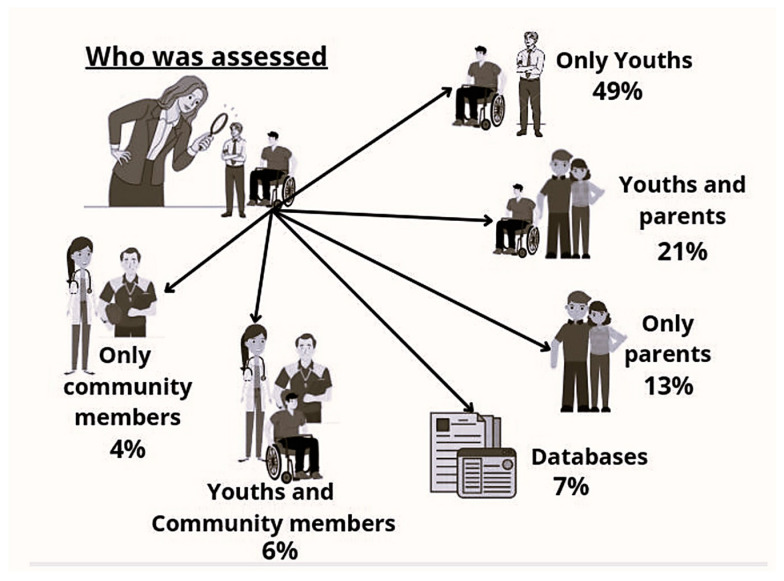
Distribution of who was assessed in the studies about AYAs with CP.

Looking at who was assessed in the included studies, we found that 42 studies (49%) assessed AYAs directly, and information from the young people and family members were collectively obtained in 18 studies, with 11 studies capturing information exclusively from a family member. Expanding the source of information, nine studies collected data from other populations such as researchers, clinicians, and teachers, and, among those, five had also heard the young people’s voices. Databases or medical records were the sources of information in nine other studies, including the reviews. Figure 3 summarizes this information.

## 4. Discussion

The transition to adult life for people with a child-onset disability can be complex, with many influencing factors needing to be better understood in order to eliminate the gap between the child and adult care received by this population. It is expected that, as youth with CP grow up, they will start to develop more independence, deal with issues that go beyond their disability, and participate more in diverse life situations. In our review, we summarized evidence to have a broader view of the current ICF focus, beyond BSF, in research exploring the health development, independence, or participation of AYAs with CP, and we also explored who is being assessed in these studies.

Looking at the research focus, the ICF component of activities, part of the AP component, was the most frequent area of interest in studies. These studies used tools or methods of assessment to analyze AYA capacities or performance in activities such as mobility [48], hand use [49], and activities of daily life [50]. Functional classification systems such as GMFCS, MACS, and CFCS are frequently used to categorize AYA capacity level or to analyze the relationship between the capacity level and other variables.

As these tools describe activity, they considerably steered the ICF linking process towards the ICF AP component. Of course, the high number of studies focusing on activities could be due to the fact that, as people with CP age, they tend to experience functional decline and other complications such as pain [51], and these aspects could impact their activity levels [52], which makes the understanding of their activity capacities or performance relevant.

Besides common barriers to participation in physical activities relating to the person’s capacity or ability to perform activities, other aspects such as social attitudes (EF), personal preferences (PF), and the confidence to do (PF) the activity are key facilitators to overcoming the barriers to participating in certain activities [53,54]. This highlights the importance of looking beyond factors related to BSF, including assessments or methods to analyze the contextual factors affecting activities that will facilitate better levels of participation for AYAs with CP.

In this sense, and also part of the ICF AP component, reviewed studies assessing participation focused on aspects such as the frequency of participation in everyday life [55], predictors (BSF and activity) of participation in domestic life, and interpersonal relationships [56]. We also explored aspects of participation relating to individual (PF) and environmental factors (EF) associated with changes in participation [57], satisfaction (PF) with the participation [58], and the association of modifiable childhood factors (PFs, EFs and BSF) on adolescents’ participation level [59].

As the transition to adulthood is a stage with significant environmental and personal changes, characteristics of activities and factors influencing participation may vary during this life period due to changes in functionality, the level of independence, and personal preferences. Thus, investigations concerning specific PFs affecting the transition process are crucial.

In our review, PFs were the second most addressed ICF component after AP, and common AYA personal variables such as age, gender, and educational level contributed to allocating PFs to this position during ICF linking. Key PFs such as AYA perspectives about the use of transition services [60], acceptance of their disability [61], and volition [62] were also explored.

It was observed that emotional support contributed to variations in the level of satisfaction with life and health status of young adults with CP [63]. PFs such as insecurity and sadness were mentioned as barriers influencing participation, while accepting the disability and having a positive attitude were facilitators according to adolescents’ perspectives [64]. Young adults with CP also emphasized the need for interventions (EF) that enable them to find their own way to perform everyday activities and be involved (AP), as this will contribute to building their personality (PF).

Along these lines, strategies aiming to enhance AYAs’ personal abilities to deal with environmental stressors or disappointments which are typical during the transition to adult life could be helpful in promoting their coping skills, empowering positive behavioural health [65], which could lead to better satisfaction with oneself, thus increasing confidence to participate and interdependent attitudes in different life situations. Facilitating this, professionals and family members could easily provide information (EF) about their disability (BSF) and future challenges (AP, PF, and EF) since the early stages of their development, enabling better levels of self-awareness and self-efficacy skills (PF) [66].

Is it possible to observe that, together with PFs, environmental factors will deeply influence our perception and development. The EFs comprise the physical, social, and attitudinal environments in which people live and conduct their lives [67]. Especially during transition, the diversity of environmental and personal changes occurring in a short period across ones lifespan brings challenges. In this sense, EFs such as accessible public places, inclusion policies for educational qualification, as well as social understandings of the capabilities of people with disabilities could be examples of easily modifiable EFs that could facilitate the transition of AYAs with disabilities. A recent review showed that EFs such as the type of school, family ecology, and parental stress were associated with participation levels for children and adolescents with CP at the beginning of their transition process [68]. Especially for young adults, governmental programs which intend to provide funding and connect people with disabilities to services in their community, such as doctors, jobs, sports, and social groups, could be a means to support a more independent, participative, and fulfilling adult life [69].

However, in our review and others concerning people with CP [19], EFs were the least focused on area among all ICF components. Among the included studies in our review, only three [43,44,45] used EFs as their exclusive research focus, and aimed to explore family members’ perspectives on healthcare needs [43,44], parental stress, and its impact on the family life [45]. Specifically, parental stress or mental health were assessed in five studies [41,45,59,70,71]. Common variables such as the parents’ educational level, family structure, and income were also frequently collected for characterization purposes, and could be used more frequently as possible influencing factors.

In the reviewed studies, family-related EFs are usually provided by a family member, but some studies explored EFs such as one’s housing situation, occupation, financial resources, and romantic relationships [17,72,73,74] through directly asking AYAs with CP. It is important to keep in mind that AYAs are at the center of the transition process, thus listening to them and asking specific questions about the different issues they experience is crucial. By letting AYAs express their own perspectives independently, their development of self-knowledge and autonomy (PF) is supported.

When using standardized tools, researchers and clinicians may face challenges in shifting the focus from parents to youth, as most of the existing tools were designed to collect information from a proxy. Expansions of these existing tools and the development of self-report versions are needed, as was done with the GMFCS [75]. It is important to think that, beyond the specific questions of standardized assessment tools, there is so much more to be explored that can help us fill the gaps in health development between childhood and adulthood.

Along these lines, our review explored who is being assessed in studies, and observed that most studies are listening primarily to the AYA. Several qualitative studies explored issues relating to health development, independence, or the participation of AYAs with CP. In these studies, the use of open-ended questions to explore young peoples’ experiences was the most common method used. Questions regarding the youths’ perspectives on their transition in health care [60], residential immersive life skill programs [76], participation [64], and lived experiences in several life areas [77] were some of the themes explored.

In studies focusing on understanding the life issues of AYAs with CP with cognitive impairments, the perspective of a person who knows the youth’s life, such as a family member, may be the only source of information possible, thus, youth’s voices need to be represented by them, as shown in Gray et al. (2021) [78].

It is important to consider that, in early adolescence, having the parents’ perspectives together with AYAs’ perspectives can be helpful in supporting them to feel more secure about the issue being asked. On the other hand, for AYAs with higher cognitive levels, the shift from asking a proxy to directly asking the youth about matters they experience is a need within research and clinical practice, as discrepancies could exist among adolescents and their relatives’ perspectives, who tend to rate adolescents’ issues more negatively [79]. These discrepancies can be natural during early and mid-adolescence, but may have more negative effects on adolescents’ wellbeing during late adolescence when they are seeking more autonomy and adapting to their health condition [80].

The transition between adolescence and adulthood involves more than just the AYA and their families, as it is expected that young people increasingly expand their connections with society during this period of life. Accordingly, some studies in our review collected information from the youth (PF) and community members (EF), such as healthcare providers, researchers, and teachers, thus providing a more complete contextual perspective.

From multiple viewpoints, Cleary and co-authors (2019) [81] explored barriers and facilitators to physical activity (AP) through listening to the adolescents, their parents, teachers, and therapists. Morris and colleagues (2018) [82] explored the factors affecting adolescents’ continuity in community-based physical activity (AP) through asking the adolescents, parents, and sports facilitators. In a knowledge translation study, perspectives from clinicians, researchers, parents, and AYAs were used to identify community-informed elements (EF) to guide the development of physical activity programs (AP) [83]. By also considering the point of view of these other figures, key information to support AYAs’ development can be collated more efficiently.

In order to evolve the understanding of facilitators and approaches to promote optimal health over the years, perhaps a successful transition between life stages could be expressed by an indicator such as QoL. QoL is not included among the ICF components, probably because it involves many related constructs that are difficult to separate clearly from each other. Additionally, the ICF creates an ‘objective’ picture of a person, but does not include subjective elements such as the self-reporting of feelings. Thus, on the ICF linking of our study, QoL was classified as ICF—not covered.

Nonetheless, from the eighty-six studies included in our review, fifteen studies assessed AYAs’ QoL, but only four studies [11,38,42,73] assessed QoL through asking the AYA in transition directly. In general, young adults with CP rated their QoL similar to the general population, but those with lower functional levels tended to score lower [73]. Similar results emerged from the study by Pagliano and co-authors (2021) [42], but, during in-depth interviews, signs of suffering due to isolation and relational difficulties were observed, aspects that had not emerged from the QoL questionnaires [42]. Thus, it is important to keep in mind that complex health issues require careful research planning so that they can be captured.

The family-centered care should be the basis of all approaches to care, but, wherever possible, it is necessary to start putting the person being cared for at the center of their own care, adopting a more person-centred approach. These processes should ideally be supported by a multidisciplinary team, who would be expected to see the AYA in transition beyond their disability, considering the several aspects that, as equally impactful as BSF-related factors, can influence a person’s development.

Based on the findings of this review, we recommend that researchers exploring health development, the independence, or the participation of AYAs with CP give greater attention to contextual factors, and, when possible, allow AYAs to speak for themselves about the issues they experience. Knowledge from this review can also support clinicians in expanding their assessment and interventional approaches when dealing with people with childhood-onset disabilities, as well as providing insights for policymakers to plan and execute new long-term policies for people with disabilities. These attitudes can contribute to bridging the gaps in knowledge, and to understanding and overcoming the barriers that interfere with the optimal health development of AYAs with CP between adolescence, adult life, and beyond.

## 5. Conclusions

This scoping review showed that, beyond BSF, studies from 2014 to 2021 exploring the health development, participation, or independence of AYAs with CP in the phase of transitioning into adulthood focused mostly on the ICF AP components, specifically on activity-related aspects. Fewer studies explored the influence of environmental factors. Most studies assessed AYAs directly mainly using qualitative studies. The perspectives of other key figures involved in their care and development were also considered. These findings emphasize the need for an expansion in the research focus towards multiple factors influencing AYAs’ health development, thus enabling clinicians to better understand and improve the care being offered to this population.

## 6. Study Limitations

No grey literature search was performed due to the high number of studies from the database search and the main focus on scientific papers only. Due to the nature of scoping reviews, the quality appraisal of included studies was not performed. The ICF linking was performed by one author; however, it was independentlychecked by at least two other authors.

## Figures and Tables

**Table 1:** List of inclusion and exclusion criteria considered during the selection process.

Inclusion Criteria	Exclusion Criteria
1—Articles published in the English language.	1—Population mean age or age range for cerebral palsy not comprised between 13 and 30 years old.
2—Between January 2014 to December 2021.	2—Studies published as “online first” and not issued in the journal between 2014–2021.
3—Main focus of interest being Cerebral palsy.	3—Studies with interventions or assessments focused only on body structures and function.
4—About adolescents and/or young adults (13–30 years old (key transition period)).	4—Studies focusing on scale validation, translation, or protocols.
5—Evaluating constructs related to the development of health, independence, or participation.	5—Documents other than scientific papers, such as theses, abstracts, expert opinions, etc.

## Data Availability

The original contributions presented in the study are included in the article/Supplementary Material; further inquiries can be directed to the corresponding author.

## Supplementary Materials

The following supporting information can be downloaded at https://www.mdpi.com/article/10.3390/ijerph21060670/s1. S1 PRISMA Checklist. PRISMA checklist for scoping reviews; S2 Appendix 1. Complete search strategy EMBASE example; S3 Appendix 2. Scoping review included studies characteristics, [84,85,86,87,88,89,90,91,92,93,94,95,96,97,98,99,100,101,102,103,104,105,106,107,108,109,110,111,112,113,114,115,116,117,118,119,120,121,122,123,124,125,126] are cited in Supplementary.
