# Supplementary material for: Looking beyond Body Structure and Function: ICF Foci and Who Is Being Assessed in Research about Adolescents and Young Adults with Cerebral Palsy—A Scoping Review
Source: Int J Environ Res Public Health. 2024 May 24;21(6):670. doi: 10.3390/ijerph21060670 (PMC11203594; doi:10.3390/ijerph21060670)
Supplement: Supplementary file 1 [file ijerph-21-00670-s001.zip › S3 Appendix 2 Studies characteristics..pdf]

Appendix 2: Studies' characteristics, with content organized according to the publication year (oldest to recent), the number of ICF components assessed (higher to lower), and the number of study participants (higher to lower).

**Author, Year, Title**	**Country**	**Study type**	**Aim**	**Participants**	**ICF components assessed / assessment method**	**Who was assessed**
**Verhoef et al. (2014) [84].**<br><br>Development of work participation in young adults with cerebral palsy: a longitudinal study	The Netherlands	Observational	To document the longitudinal development of work participation in young adults with CP during their transition to adulthood over a 4-year period. A further aim was to examine the associations of demographic and clinical characteristics with work participation in young adults with CP who were in the age range 20–24 years. Finally, we aimed to determine experienced work limitations and situational or health barriers to employment among employed persons.	Total n= 74<br>CP= 74<br>Other= 0<br><br>Mean age (CP): 22.8 years<br><br>GMFCS: I-V<br>MACS: I-V	*Activity:* work participation, employment situation (type of job, work hours) and occupational disability; Work Limitations Questionnaire.<br><br>*Personal Factors:* age, gender, level of education, General Self-Efficacy Scale-12 (GSES-12).<br><br>*Environmental factors:* parents' level of education.<br><br>*YCF Not-defined:* Amenities and Services Utilization Survey (AVO).<br><br>*Body function:* type and laterality of Cerebral Palsy (CP), gross motor function, manual ability.	Young adult

**Alriksson-Schmidt et al. (2014) [72].**<br><br>Follow-up of individuals with cerebral palsy through the transition years and description of adult life: the Swedish experience	Sweden	Descriptive cross-sectional	To describe the process of providing healthcare through the transition years to individuals with cerebral palsy (CP) and to present data on living arrangements, education/occupation status, and use of personal assistance in young Swedish adults with CP.	Total n= 102<br>CP= 102<br>Other= 0<br><br>Mean age (CP): 20.6 years<br><br>GMFCS: I-V<br>MACS: I-V	*Activity:* GMFCS, MACS.<br><br>*Environmental factors:* participants' living arrangements; personal assistance, Occupation.	Young adult
**Ben-Pazi et al. (2014) [85].**<br><br>Can global positioning systems quantify participation in cerebral palsy?	Israel	Observational	To examine whether motor-related participation could be assessed by global positioning systems in individuals with cerebral palsy.	Total n= 2<br>CP= 1<br>Other= 1<br><br>Mean age (CP):<br>14.5 years<br><br>Functional classification not reported.	*Activity:* Positioning logger device.<br><br>*Participation:* Children's Assessment of Participation and Enjoyment (CAPE).	Adolescent /Parent

**Blackman & Conaway, (2014) [43].**<br><br>Adolescents with cerebral palsy: transitioning to adult health care services.	The United States of America	Retrospective	To seek answers to the following questions: (a) What are the diagnosed health, developmental, and emotional comorbidities of adolescents with CP, compared with those of adolescents with a chronic, but not disabling, health condition? (b) How do parents rate the functional difficulties of adolescents with CP? (c) How well are adolescents with CP preparing for transition to adult health care services?	Total n= 4038<br>CP= 80<br>Other= 3894<br><br>Age range (CP): 15-17 years<br><br>Functional classification not reported.	*Environmental factors:* National Survey of Children with Special Health Care Needs (NS-CSHCN).	Parent/ Caretakers
**Michelsen et al. (2014) [55].**<br><br>European study of frequency of participation of adolescents with and without cerebral palsy.	Denmark	Longitudinal	To compare the frequency of participation in everyday life of adolescents across the spectrum of severity of CP and adolescents in the the general population in nine European regions.	Total n= 667<br>CP= 667<br>Other= 0<br><br>Mean age: 14.6 years<br><br>GMFCS: I-V	*Participation:* Questionnaire of Young People Participation.<br><br>*Body function:* Severity of impairment; Motor impairment; GMFCS; intellectual impairment as estimated IQ.	Adolescent

**Nooijen et al. (2014) [86].**<br><br>Inactive and sedentary lifestyles amongst ambulatory adolescents and young adults with cerebral palsy.	The Netherlands	Longitudinal, randomised controlled trial	To assess physical behavior, including physical activity and sedentary behaviour, of ambulatory adolescents and young adults with cerebral palsy (CP)	Total n= 80<br>CP= 48<br>Other= 32<br><br>Mean age: 20 Years<br><br>GMFCS: I-III	*Activity:* Physical Activity Scale for Individuals with Physical Disabilities (PASIPD)/ Accelerometer.	Adolescent /Young adult
**Slaman et al. (2015) [38].**<br><br>A lifestyle intervention improves fatigue, mental health and social support among adolescents and young adults with cerebral palsy: focus on mediating effects.	The Netherlands	Randomized controlled trial	To evaluate the effect of a lifestyle intervention on fatigue, participation, quality of life, gross motor functioning, motivation, self-efficacy and social support, and to explore mediating effects of physical behavior and physical fitness.	Total n= 57<br>CP= 57<br>Other= 0<br><br>Mean age: 20 Years<br><br>GMFCS: I-V	*Activity:* Gross motor function measure (GMFM-66) / self reported activity data.<br><br>*Participation:* Life Habits Questionnaire (LIFE-H 3.0).<br><br>*Personal Factors:* General Self -efficacy scale (GSES).<br><br>*Environmental factors:* Social Support for Exercise Behaviour Scale (SSEBS).<br><br>*ICF Not-Covered:* Short-Form health survey (SF-36).<br><br>Body function: fatigue Severity Scale (FSS); Checklist Individual Strength (fatigue item); peak oxygen uptake.	Adolescent

**Colver et al. (2015) [10].**<br><br>Self-reported quality of life of adolescents with cerebral palsy: a cross-sectional and longitudinal analysis.	The United Kingdom	longitudinal follow-up and cross-sectional analysis	To assess whether QoL changes between childhood and adolescence, and whether QoL in adolescence is predicted by QoL in childhood, pain in childhood and adolescence, psychological problems and parenting stress in childhood, and their changes between childhood and adolescence.	Total n= 431<br>CP= 431<br>Other= 0<br><br>Mean age: 15.1 years<br><br>GMFCS: I-V	*Activity:* GMFCS, fine motor function, seizures, feeding, communication<br><br>*Personal Factors:* Strengths and Difficulties Questionnaire (SDQ).<br><br>*Environmental factors:* family structure, and parents' educational qualifications<br><br>YCF-not covered: Health Questionnaire for Children and Young People (KIDSCREEN).<br><br>---<br>*Body function:* type of cerebral palsy, intellectual ability.	Adolescent /Parent
**Dang et al. (2015) [59].**<br><br>Predictors of participation of adolescents with cerebral palsy: A European multi-centre longitudinal .	France	Not clearly specified	To evaluate how participation of adolescents with CP is associated with modifiable childhood factors: pain, psychological problems, and parenting stress. We studied whether these associations were mediated by participation in childhood or by the level of the same predictors in adolescence.	Total n= 818<br>CP= 818<br>Other= 0<br><br>Mean age: 15.1 years<br><br>GMFCS: I-V	*Activity:* GMFCS, Bimanual Fine Motor Function (BFMF), feeding, communication.<br><br>*Participation:* questionnaire of Life Habits (LIFE-H).<br><br>*Environmental factors:* family structure, parents' educational qualifications, parent stress.<br><br>---<br>*Body function:* pain; seizures, intellectual ability.	Adolescent /Parents

**Slaman et al. (2015) [87].**<br><br>Cost-utility of a lifestyle intervention in adolescents and young adults with spastic cerebral palsy.	The Netherlands	Randomized controlled trial	To estimate the cost-utility of a lifestyle intervention compared with usual care in adolescents and young adults with spastic CP.	Total n= 57<br>CP= 57<br>Other= 0<br><br>Mean age: 20 years<br><br>GMFCS: I-IV	*Activity:* levels of physical activity and physical fitness.<br><br>*ICF not-defined:* Cost-utility of a lifestyle intervention.<br><br>*ICF not-covered:* Quality of life (SF-36).	Adolescent /Rehabilitation Physician/ Healthcare Providers
**Usuba et al. (2015) [88].**<br><br>Leisure-Time Physical Activity in adults with Cerebral Palsy.	Canada	Cross-sectional, follow-up-survey	The primary purpose of this study was to describe the level of participation in LTPAs in adults with CP in terms of frequency and intensity. A secondary purpose was to provide an exploratory analysis of the variations in LTPA in adults with CP attributable to ambulatory status.	Total n= 2785<br>CP= 54<br>Other= 2731<br><br>Mean age (CP): 29.5 years<br><br>GMFCS: I-V	*Activity:* Leisure-Time Physical Activity (LTPA-activities related questions); GMFCS.<br><br>*Personal Factors:* participants' background, gender, age, living situation, living with, marital status, employment, and personal income.	Young adult

**McDowell et al. (2015) [89].**<br><br>Service use and family-centred care in young people with severe cerebral palsy: a population-based, cross-sectional clinical survey.	The United Kingdom	Prospective, cross-sectional survey	the aims of this paper are twofold; (i) to report on service use in a representative series of children and young adults with severe forms of CP and (ii) to determine the extent to which families perceive their services to be family-centred, with a view to making recommendations for future service development and improvement.	Total n= 123<br>CP= 123<br>Other= 0<br><br>Mean age: 16.2 years<br><br>GMFCS: IV-V	*Activity:* GMFCS.<br><br>*Environmental factors:* schooling, aids/adaptations; service provision; Measure of Processes of Care-20 (MPOC-20).<br><br>Body function: Intellectual impairment; associated impairments	Parents
**Kenyon et al. (2015) [47].**<br><br>Promoting self-exploration and function through an individualized power mobility training program.	The United States of America	Interventional	To describe the development and implementation of an intervention program that used a Power Wheelchair Trainer (Trainer) to enable an individual with severe impairments to participate in power mobility training.	Total n= 1<br>CP= 1<br>Other= 0<br><br>Age: 18 years<br><br>GMFCS: V<br>MACS: V<br>CFCS: V	*Activity:* functional motor skills; Gross motor function measure(GMFM), Child Health Index of Life with Disabilities (CPCHILD).<br><br>*Environmental factors:* power mobility device.<br><br>*Body function:* active head/trunk control.<br><br>*Body structure:* range of motion of the upper and lower extremities.	Adolescent / Parent

**Ramstad et al. (2015) [79].**<br><br>Self-reported mental health in youth with cerebral palsy and associations to recurrent musculoskeletal pain.	Norway	Cross-sectional	To explore self-reports on emotional, conduct, hyperactivity and peer problems and on prosocial behavior in youth with cerebral palsy (CP), compare the reports to normative data and to mothers' reports and to analyse if recurrent CP-related musculoskeletal pain (RMP) influence the self-reports.	Total n= 81<br>CP= 81<br>Other= 0<br><br>Mean age: 14.2 years<br><br>GMFCS: I-V	*Personal Factors:* Strengths and Difficulties Questionnaire (SDQ).<br><br>*Body function:* pain.	Adolescent / Parent
**Vles et al. (2015) [90].**<br><br>Quality of Life of Children with Cerebral Palsy: A Cross-Sectional KIDSCREEN study in the Southern part of the Netherlands.	The Netherlands	Cross-sectional study	To compare the quality of life (QoL) of 8-18 years old children with cerebral palsy (CP) in the Southern part of The Netherlands to a sample of European children from the general population and to investigate factors associated with possible differences.	Total n= 80<br>CP= 80<br>Other= 0<br><br>Mean age: 13.4 years<br><br>Functional classifications not reported.	*YCF Not-covered:* Health Questionnaire for Children and Young People (KIDSCREEN).	Parents

**Majnemer et al. (2015) [57].**<br><br>Stability of leisure participation from school-age to adolescence in individuals with cerebral palsy.	Canada	Prospective study	To describe changes in leisure participation and leisure preferences from school age (6–12 years) to adolescence (12–19 years) in individuals with CP. This study also explored individual and environmental factors that may be associated with any changes in participation over time.	Total n= 38<br>CP= 38<br>Other= 0<br><br>Mean age: 15 years<br><br>GMFCS: I-II	*Participation: Children's Assessment of Participation and Enjoyment* (CAPE); Preferences for Activities of Children (PAC).	Adolescent
**Slaman et al. (2015) [91].**<br><br>Can a lifestyle intervention programme improve physical behaviour among adolescents and young adults with spastic cerebral palsy?	The Netherlands	Randomized controlled trial	To evaluate the effectiveness of a lifestyle intervention programme on physical behavior.	Total n= 36<br>CP= 36<br>Other= 0<br><br>Mean age: 20 years<br><br>GMFCS: I-IV	*Activity:* Walking monitor / Physical Activity Scale for Individuals with Physical Disabilities (PASIPD).	Young adult

**Chikwanha et al. (2015) [44].**<br><br>Occupational therapy needs of adolescents and young adults with cerebral palsy in Zimbabwe: caregivers' perspectives.	Zimbabwe	Descriptive cross-sectional research	To determine the occupational therapy needs of adolescents and young adults with cerebral palsy living in an urban setting, to identify participation levels in activities of daily living and social activities, to identify factors influencing participation and to determine the contribution of occupational therapy in participating in these activities.	Total n= 35<br>CP= 0<br>Other= 35<br><br>Age range: 12-25 years<br><br>Functional classification not reported.	*Environmental factors:* Open question regarding caregivers' perspectives about occupational therapy needs.	Caregiver
**Hynan et al. (2015) [92].**<br><br>A grounded theory of Internet and social media use by young people who use augmentative and alternative communication (AAC).	The United Kingdom	Qualitative	To know the views of young people who use AAC about their use of the Internet and social media, their perceptions of accessibility, and the potential impact upon attitudes to self representation and social ties.	Total n= 25<br>CP= 25<br>Other= 0<br><br>Mean age: 20 years<br><br>Functional classification not reported.	*Personal Factors:* Open questions regarding participants' view about Internet and social media use.	Young adult

**Bantjes et al. (2015) [93].**<br><br>When they call me cripple: a group of South African adolescents with cerebral palsy attending a special needs school talk about being disabled.	South Africa	Qualitative	To understand what it is like for participants to be disabled and how they experience their bodies.	Total n= 15<br>CP= 15<br>Other= 0<br><br>Mean age: 14.4 years<br><br>Functional classification not reported.	*Personal Factors:* Open question regarding how it is Participants' experiences/views about being disable. They were asked to describe their disability and their understanding of having CP. They were invited to share their experiences and express their hopes for the future.	Adolescent

**Bantjes et al. (2015) [94].**<br><br>Developing programmes to promote participation in sport among adolescents with disabilities: Perceptions expressed by a group of South African adolescents with cerebral palsy.	South Africa	Qualitative	To understand what a group of adolescents with cerebral palsy (CP) living in South Africa perceive to be important components of programs developed to increase their participation in sport.	Total n= 15<br>CP= 15<br>Other= 0<br><br>Age range: 12-18 years<br><br>Functional classification not reported.	*Personal Factors:* Open question regarding participants' lived experience of physical activity.	Adolescent

**Björquist et al. (2015) [95].**<br><br>Living in transition - experiences of health and well-being and the needs of adolescents with cerebral palsy.	Sweden	Inductive qualitative approach	To gain a deeper understanding of how adolescents with CP experience their own health, well-being and needs of support during their transition to adulthood.	Total n= 12<br>CP= 12<br>Other= 0<br><br>Age range: 17-18 years<br><br>Funcional claasificatio n not reported.	*Personal Factors:* Open question about adolescents' perspectives about life at the present moment when they are young and soon to become adults.	Adolescent
**Carroll et al. (2015) [60].**<br><br>Health Care Transition Experiences of Young Adults With Cerebral Palsy.	The United States of America	Phenomenological Method	To uncover the meaning of transition to adult-centered care as experienced by YA-CP participants.	Total n= 9<br>CP= 9<br>Other= 0<br><br>Age range: 19-25 years<br><br>Functional classificatio n not reported.	*Personal Factors:* Open question regarding how participants' Health Care Transition Experiences.	Young adult

**Koldoff et al. (2015) [96].**<br><br>Physical Activity Among Adolescents with Cerebral Palsy: An Integrative Review.	The United States of America	Review	An integrative review was performed to determine the state of the science regarding 1) what factors impact physical activity in adolescents with cerebral palsy, and 2) how the needs of this population have been addressed regarding physical activity	Total n= diverse<br>CP= diverse<br>Other= diverse<br><br>Mean age: diverse<br><br>Functional classification: diverse	*Activity:* mobility or activity aspects.	Database
**Jiang et al. (2016) [97].**<br><br>Quality of life in young adults with cerebral palsy.	Australia	Cross-sectional study	The primary aim of this study was to compare the QOL of a cohort of young Australian adults with CP with a cohort of able-bodied peers, using the same multidimensional instrument. To gain insight into the determinants of QOL, a further aim was to explore the relationship between QOL and impairments, functioning, and social participation.	Total n= 335<br>CP= 335<br>Other= 0<br><br>Mean age: 24.7 years<br><br>GMFCS: I-V	*Activity:* Barthel Index; GMFCS.<br><br>*Participation:* social participation.<br><br>*Environmental factors:* personal income.<br><br>*ICF not-covered:* Quality of Life Instrument for Young Adults (YAQOL).<br><br>---<br><br>*Body function:* CP subtype, comorbidities, intellectual, speech, hearing, and vision impairments.	Young adult/ Parent/ Medical records

**Eken et al. (2016) [98].**<br><br>Relations between muscle endurance and subjectively reported fatigue, walking capacity, and participation in mildly affected adolescents with cerebral palsy.	The Netherlands	Case–control study	The primary aim of this study was to establish whether adolescents with CP more frequently report fatigue as a problem than peers with typical development. The second aim was to investigate the relation between muscle endurance of the knee extensors and subjectively reported fatigue, walking capacity, and participation in adolescents with CP.	Total n= 35<br>CP= 17<br>Other= 18<br><br>Mean age (CP): 15.9 years<br><br>Age range (Other): 13-19 years<br><br>GMFCS: I-II	*Participation:* Life Habits Questionnaire (Life-H).<br><br>*Body function:* Muscle endurance; Fatigue (PedsQL); walking (6MWT).	Adolescent

**Conchar et al. (2016) [99].**<br><br>Barriers and facilitators to participation in physical activity: The experiences of a group of South African adolescents with cerebral palsy.	South Africa	Qualitative	To investigate the lived experiences of a group of South African adolescents with cerebral palsy (CP), and their involvement in physical activity, in order to better understand their experience of physical activity and the individual, social and contextual factors that hinder and promote their participation.	Total n= 15<br>CP= 15<br>Other= 0<br><br>Age range: 12-18 years<br><br>Funcional claasification not reported.	*Personal Factors:* Open question about how sports programs could be designed to promote participation in health-promoting physical activities.	Adolescent
**Wright et al. (2016) [100].**<br><br>Transition from paediatric to adult health services in Scotland for young people with cerebral palsy.	The United Kingdom	Qualitative	To investigate community pediatricians' perceptions of current services for transition from paediatric to adult health care for young people with CP and how these might be improved.	Total n= 13<br>CP= 0<br>Other= 13<br><br>Mean age: NR<br><br>Functional classification: not aplicable.	*Personal factors:* Open question related to paediatricians' perceptions of current services for transition.	Healthcare professional

**Mumford & Chau, (2016) [101].**<br><br>Application of an access technology delivery protocol to two children with cerebral palsy.	Canada	Mixed methods case studies	To delineates the merits and limitations of the Access Technology Delivery Protocol (ATDP) through its application to two children with severe disabilities.	Total n= 2<br>CP= 2<br>Other= 0<br><br>Mean age: 13 years<br><br>GMFCS: V	*ICF not-defined:* access a technology delivery protocol.	Adolescent
**Rapp et al. (2017) [70].**<br><br>Predictors of parent-reported quality of life of adolescents with cerebral palsy: A longitudinal study.	Germany	Longitidinal cohort study	To understand if 1) parent-reported QoL of adolescents with CP changes as young people move from childhood to adolescence.<br>2) QoL in adolescence is predicted by:<br>a) childhood factors (impairment, pain, psychological problems),<br>b) family and personal factors of the reporting parent (socio-economic and parenting stress),<br>c) changes in pain, psychological problems and parenting stress between childhood and adolescence.	Total n= 551<br>CP= 551<br>Other= 0<br><br>Mean age: 15.1 years<br><br>GMFCS: I-V	*Activity:* GMFCS, Bimanual Fine Motor Function (BFMF), feeding, communication.<br><br>*Personal factors:* Strengths and Difficulties Questionnaire (SDQ).<br><br>*Environmental factors:* family structure and parents' educational qualifications, Parenting Stress Index Short Form (PSI).<br><br>*ICF not-covered*: Health Questionnaire for Children and Young People (KIDSCREEN).<br><br>*Body function:* seizures, intellectual ability.	Parent

**Monbaliu et al. (2017) [102].**<br><br>The relationship of dystonia and choreoathetosis with activity, participation and quality of life in children and youth with dyskinetic cerebral palsy.	Belgium	Cross-sectional study	To relate dystonia and choreoathetosis with activity, participation and quality of life (QOL) in children and youth with dyskinetic Cerebral Palsy (CP).	Total n= 54<br>CP= 54<br>Other= 0<br><br>Mean age: 14.6 years<br><br>GMFCS: I-V<br>MACS: I-V	*Activity:* GMFM, Functional Mobility Scale (FMS), the Jebsen-Taylor Hand Function Test (JTT), the ABILHAND-Kids Questionnaire (ABIL-K).<br><br>*Participation:* Life Habits Questionnaire (LIFE-H).<br><br>*ICF not-covered:* Quality of Life Questionnaire for children with CP (CP-QOL).<br>*Body function:* Dyskinesia Impairment Scale.	Adolescent /Parent

**Frank & Souza, (2017) [103].**<br><br>Problematic clinical features of children and adults with cerebral palsy who use electric powered indoor/outdoor wheelchairs: A cross-sectional study.	The United Kingdom	Cross-sectional retrospective review study	To describe the clinical features of electric-powered indoor/outdoor wheelchair (EPIOC) users with cerebral palsy (CP) that are problematic to optimal prescription and to explore comorbidities, features of CP, and conditions secondary to disability impacting on equipment provision for children and adults.	Total n= 102<br>CP= 102<br>Other= 0<br><br>Mean age: 28.6 years<br><br>GMFCS: IV-V	*Personal factors:* age and gender<br><br>*Environmental factors:* wheelchair factors (TIS, cushions, and complex controls).<br><br>*Body function:* comorbidities, complications relating to CP, or disability.	Medical records

**Teixeira-Machado et al. (2017) [50].**<br><br>Dance Improves Functionality and Psychosocial Adjustment in Cerebral Palsy: A Randomized Controlled Clinical Trial.	Brazil	Randomized controlled clinical trial	To investigate the effects of dance in functionality, independence function, social life, and psychosocial adjustments in CP young subjects.	Total n= 26<br>CP= 26<br>Other= 0<br><br>Mean age: 17.8 years<br><br>GMFCS: II-V	*Activity:* World Health Organization Disability Assessment Schedule (WHODAS), Functional Independence Measure (FIM); GMFCS.<br><br>*Personal factors:* demographic data.	Young adult
**Bagatell et al. (2017) [15].**<br><br>"Thrust into adulthood": Transition experiences of young adults with cerebral palsy.	The United States of America	Descriptive qualitative study	To explore the transition experiences, perceptions, and needs of young adults with cerebral palsy living in one state in the southeastern United States.	Total n= 9<br>CP= 9<br>Other= 0<br><br>Mean age: 21.2 years<br><br>GMFCS: I-V	*Activity:* GMFCS.<br><br>*Personal factors:* Open questions related to participants' perspectives about transition programs/process.	Young adult

**Guyard et al. (2017) [45].**<br><br>Family adaptation to cerebral palsy in adolescents: A European multicenter study.	France	Cross-sectional	To describe the family adaptation to disability based on a conceptual model related to the ABCX model in order to identify determinants associated with the level of family adaptation in families living with an adolescent with cerebral palsy (CP).	Total n= 286<br>CP= 286<br>Other= 0<br><br>Mean age (CP): 15.1 years<br><br>GMFCS: IV-V	*Environmental factors:* parental distress, perceived impact in various dimensions of family life, family resources and stressors.	Family member
**Gorter et al. (2017) [83].**<br><br>Consensus Planning Toward a Community-Based Approach to Promote Physical Activity in Youth with Cerebral Palsy.	Canada	Integrative Knowledge Translation	To explore knowledge translation initiatives to identify community-informed elements to guide the development of an optimal physical activity program for youth with cerebral palsy (CP) and to support research efforts.	Total n= 137<br>CP= NR<br>Other= 137<br><br>Mean age: NR<br><br>Functional classification not reported.	*Personal factors:* clinicians/researchers/parents/youth perspectives about health services.	Clinicians/ Researchers/Parents/ Youth

**Voyer et al. (2017) [104].**<br><br>Sociometric status and the attribution of intentions in a sample of adolescents with cerebral palsy.	Canada	Longitudinal	The objective of this study was, first, to confirm the negative sociometric status of children with CP compared with children without CP at age 10, and second, to examine the longitudinal pattern that might explain the development of a cognitive bias (described as a percentage of hostile AI) at age 15.	Total n= 117<br>CP= 60<br>Other= 57<br><br>Mean age (CP): 15 years<br><br>Mean age (Other): 14.8 years<br><br>GMFCS: I-II	*Personal factors:* Open question (sociometric interview) related to participants' perspectives/experiences in socialization.	Adolescent
**Caron & Light, (2017) [105].**<br><br>Social media experiences of adolescents and young adults with cerebral palsy who use augmentative and alternative communication.	The United States of America	Qualitative	To expand the current understanding of how adolescents and young adults with cerebral palsy (CP) and complex communication needs use social media.	Total n= 7<br>CP= 7<br>Other= 0<br><br>Mean age: 18 years<br><br>Functional classification not reported.	*Personal factors:* Open questions related to participants' experiences using social media.	Adolescent

**Koldoff, (2017) [106].**<br><br>Parental perspectives on physical activity of adolescents with cerebral palsy: a pilot study.	The United States of America	Qualitative descriptive	1. To explore a parent's perspective about physical activities that their adolescent has experienced. 2. To investigate parental attitudes and beliefs about the importance of physical activity for their child. 3. To describe a parent's viewpoint about facilitators/barriers of physical activity for teens with disability.	Total n= 5<br>CP= 5<br>Other= 0<br><br>Age range: 19-30 years<br><br>GMFCS: I-III<br>MACS: I-II<br>CFCS: I-II	*Personal factors:* adolescents' demographic information; parents' perspectives about adolescent's experience with physical activity.	Parents
**Liljenquist et al. (2018) [37].**<br><br>Utilization of Physical Therapy Services During Transition for Young People With Cerebral Palsy: A Call for Improved Care Into Adulthood.	The United States of America	Secondary analysis of the National Longitudinal Transition Study 2.	To identify factors contributing to the utilization of physical therapy services for youth with CP both during and after secondary school.	Total n= 35.290<br>CP= 35.290<br>Other= 0<br><br>Age range: 13-26 years<br><br>GMFCS: II-V<br>MACS: II-V	*Activity:* Use of a mobility device, Typical use of hands and arms, Communication problem.<br><br>*Personal factors:* Sex, race.<br><br>*Environmental factors:* Parent education level, general coordination of care, indication of access to a medical home, General Access to Disability Support Services, Receipt of physical therapy. General access to disability support services, Poverty line status.<br>---<br>*Body function:* Second disability	Youth, Parents and Guardians, Teachers, Principals, School records.

**Sienko et al. (2018) [63].**<br><br>An exploratory study investigating the multidimensional factors impacting the health and well-being of young adults with cerebral palsy.	The United States of America	Cross-sectional	To examine the ambulatory decline, pain, pain interference, depression, fatigue, locus of control, emotional support, overall health status and satisfaction with life of young adults with cerebral palsy, age 18–30 years.	Total n= 97<br>CP= 97<br>Other= 0<br><br>Mean age: 23.8 years<br><br>GMFCS: I-V	*Activity:* GMFCS.<br><br>*Participation:* Impact of Pain on Functioning and Well-being.<br><br>*Personal factors:* Health and well-being, Emotional support, health status and satisfaction with life.<br><br>*Body function:* pain, Fatigue, depression	Adolescent

**Morris et al. (2018) [82].**<br><br>Sustained participation in community-based physical activity by adolescents with cerebral palsy: a qualitative study.	Australia	Qualitative	To identify what factors contribute to sustained participation in physical activity by adolescents with cerebral palsy from the perspectives of the adolescents, their parents, and their community activity facilitator.	Total n= 15<br>CP= 5<br>Other= 10<br><br>Mean age (CP): 14.6 years<br><br>Age range (Other): 25-64 years<br><br>GMFCS: I-III	*Activity:* frequency and length of time in activity participation.<br><br>*Personal factors:* age, gender, adolescents perception about on what helped them to sustain their participation in physical activity.<br><br>*Environmental factors:* socio-economic information, facilitator's qualifications and their role in the adolescent's physical activity, parents perspectives about how the adolescent started in the activity, and what helped them to continue their participation, facilitators perception about the influence of their role, and the activity environment, such as the sports club.	Adolescent /Parent/ Sports Facilitator

**Freeman et al. (2018) [16].**<br><br>"If I had been given that information back then": An interpretive description exploring the information needs of adults with cerebral palsy looking back on their transition to adulthood.	Canada	Qualitative	To ask adults with CP between the ages of 20 and 40 to reflect on information they received during the transition to adulthood	Total n= 9<br>CP= 9<br>Other= 0<br><br>Mean age: 29.8 years<br><br>GMFCS: II-IV	*Activity:* GMFCS<br><br>*Participation:* Rotterdam Transition Profile (RTP).<br><br>*Personal factors:* Participants' reflections about participation experiences.	Young adult

**Michalska et al. (2018) [107].**<br><br>Quality of life in children and young people with tetraplegic cerebral palsy.	Poland	Descriptive study	To describe HRQOL of children and young people with tetraplegic cerebral palsy from the parents' perspectives, and explore the impact of four factors (severity of CP, intellectual disability, age, and gender) on HRQOL.	Total n= 149<br>CP= 149<br>Other= 0<br><br>Mean age: 13 years<br><br>GMFCS: II-V<br>MACS: II-V	*Activity:* GMFCS, MACS.<br><br>*ICF not-covered:* health-related quality of life (HRQOL).	Parents/ Caregivers
**King et al. (2018) [108].**<br><br>Youth Engagement in Pediatric Rehabilitation: Service Providers' Perceptions in a Real-Time Study of Solution-Focused Coaching for Participation Goals.	Canada	Interventional	The first objective was to describe patterns of engagement for youth with cerebral palsy over the course of an intervention targeting participation-oriented goals, using the PRIME-SP. The second objective was to examine the engagement components and related factors noted by service providers on a session by session basis when completing an open-ended question on the PRIME-SP. The third objective was to explore relationships among engagement, related factors, and youth outcomes, as measured by the Canadian Occupational Performance Measure (COPM) and goal attainment scaling (GAS).	Total n= 10<br>CP= 10<br>Other= 0<br><br>Mean age: 17 years<br><br>Functional classification not reported	*Participation:* Canadian Occupational Performance Measure (COPM), goal attainment scaling (GAS).<br><br>*Environmental factors:* Pediatric Rehabilitation Intervention Measure of Engagement—Service Provider version (PRIME-SP).	Service provider

**Wintels et al. (2018) [64].** How do adolescents with cerebral palsy participate? Learning from their personal experiences.	The Netherlands	Qualitative	To examine the personal participation experiences of adolescents (aged 12-17 years) with CP.	Total n= 23 CP= 23 Other= 0 Mean age: 15.3 years GMFCS: I-V CFCS: I-V	Personal factors: adolescents reflected and share their participation experiences in several daily life areas (school, sports, health care and work).	young adult
**Taherian & Davies, (2018) [109].** Multiple stakeholder perceptions of assistive technology for individuals with cerebral palsy in New Zealand.	New Zealand	Qualitative	To gain an understanding of the experiences and perspectives of assistive technology from different stakeholders in technology adoption, in the New Zealand context.	Total n= 13 CP= 5 Other= 8 Mean age (CP): 23.7 years Age range (Other): 20-59 years GMFCS: I-II	*Personal factors:* Open question related to adolescent/caregiver/health professionals perceptions about current assistive technology (AT) use.	Adolescent /Caregiver/ Health professional

**Freeman et al. (2018) [110].**<br><br>Information needs of young people with cerebral palsy and their families during the transition to adulthood: a scoping review.	Canada	Scoping review	To map the current published evidence within the transition to adulthood literature to explore what is known about information needs during the transition to adulthood of young people with CP and their parents.	Total n= 10<br>CP= 10<br>Other= 0<br><br>Mean age: 17 years<br><br>Funcional classification: diverse	*ICF not-defined:* papers referencing the transition to adulthood, CP, and information needs or provision.	Databases
**Smits et al. (2019) [39].**<br><br>Participation in Social Roles of Adolescents With Cerebral Palsy: Exploring Accomplishment and Satisfaction.	The Netherlands	Cohort study/longitudinal analysis	To explore participation in social roles of adolescents (aged 12-18 years) with cerebral palsy (CP), in terms of satisfaction compared to accomplishment.	Total n= 45<br>CP= 45<br>Other= 0<br><br>Mean age: 15.6 years<br><br>GMFCS: I-V<br>MACS: I-V<br>CFCS: I-V	*Activity:* Pediatric Evaluation of Disability Inventory (PEDI), GMFM-66; GMFCS; MACS.<br><br>*Participation:* Life Habits Questionnaire (Life-H).<br><br>*Personal factors:* Child Behavior Checklist.<br><br>*Environmental factors:* Pediatric Evaluation of Disability Inventory-Caregiver scale (PEDI-CAS); parental level of education; Utrecht Coping List (UCL).<br><br>*Body function:* prematurity	Adolescent

**Burak & Kavlak, (2019) [111].**<br><br>Investigation of the relationship between quality of life, activity participation and environmental factors in adolescents with cerebral palsy.	Turkey	Not clearly specified	To show the relationship between quality of life, activity participation and environmental factors in adolescents with CP	Total n= 75<br>CP= 75<br>Other= 0<br><br>Mean age (CP): 15.5 years<br><br>GMFCS: I-III<br>MACS: I-III	*Activity:* GMFCS, MACS, CFCS<br><br>*Personal factors:* name, gender, age, height, medical histories<br><br>*Environmental factors:* using of walking aids<br><br>*ICF not-defined:* International Classification of Functioning, Disability and Health Child-Youth version Short Form (ICF-CY)<br><br>*ICF not-covered:* Pediatric Quality of Life Inventory<br><br>*Body function:* clinical type, limb involvement, weight, Body Mass Index (BMI)	Adolescent /Parent
**Sienko, (2019) [53].**<br><br>Understanding the factors that impact the participation in physical activity and recreation in young adults with cerebral palsy (CP).	The United States of America	Cross-sectional	To examine the role of functional motor level and environmental barriers on participation in physical activity and recreation in young adults with CP.	Total n= 97<br>CP= 97<br>Other= 0<br><br>Mean age (CP): 23.8 years<br><br>GMFCS: I-V	*Activity:* Physical Activity Scale for Individuals with Physical Disabilities (PASIPD); GMFCS.<br><br>*Participation:* Life Habits Questionnaire (LIFE-H).<br><br>*Environmental factors:* Craig Hospital Inventory of Environmental Factors (CHIEF).	Young adult

**Jacobson et al. (2019) [112].**<br><br>Exploring social participation in young adults with cerebral palsy.	Sweden	Cross-sectional study	To describe a set of social outcomes in young adults with CP, using a population-based approach. The second aim was to explore associations between the social outcomes with the classification systems GMFCS, MACS, CFCS, and intellectual disability.	Total n= 61<br>CP= 61<br>Other= 0<br><br>Mean age: 21.2 years<br><br>GMFCS: I-V<br>MACS: I-V<br>CFCS: I-V	*Activity:* GMFCS, MACS, CFCS.<br><br>*Personal factors:* intellectual disability, country of birth.<br><br>*Environmental factors:* living arrangements, occupation, personal finances, support with activities of daily living, relationships, parental level of education and parental social status.	Young adult/ parent
**Power et al. (2019) [113].**<br><br>Health-related quality of life and mental health of adolescents with cerebral palsy in rural Bangladesh.	Australia	Case-control study	To assess the health-related quality of life (HRQoL) and mental health of adolescents with cerebral palsy (CP) in rural Bangladesh	Total n= 327<br>CP= 154<br>Other= 173<br><br>Mean age (CP/Other): 15.1/14.9 years<br><br>GMFCS: I-V	*Personal factors:* Strengths and Difficulties Questionnaire (SDQ).<br><br>*ICF not-covered:* Cerebral Palsy Quality of Life-Teens proxy report questionnaire (CPQoL-Teens), Health Questionnaire for Children and Young People (KIDSCREEN-27).	Adolescent /Parent

**van Gorp et al. (2019) [56].**<br><br>Long-term course of difficulty in participation of individuals with cerebral palsy aged 16 to 34 years: a prospective cohort study.	The Netherlands	Long-term prospective cohort study	To examine the long-term course of difficulty in participation of individuals with CP in GMFCS levels I to IV (without intellectual disability) aged 16 to 34 years.	Total n= 151<br>CP= 151<br>Other= 0<br><br>Mean age: 22.6 years<br><br>GMFCS: I-V	*Activity:* GMFCS<br><br>*Participation:* Life Habits Questionnaire (Life-H).<br><br>*Body function:* Type of motor impairment.	Youth
**Cleary et al. (2019) [81].**<br><br>Barriers to and facilitators of physical activity for children with cerebral palsy in special education.	Australia	Descriptive study using qualitative methods	To explore the barriers to and facilitators of physical activity for young people with cerebral palsy in specialist schools	Total n= 73<br>CP= 10<br>Other= 63<br><br>Mean age (CP/Other): 14.1 years / not reported<br><br>GMFCS: I-V	*Activity:* GMFCS.<br><br>*Personal factors:* Participants' perspectives about physical activity.	Adolescent, Parent, Teachers, Therapist

**Remijn et al. (2019) [114].** "Everyone sees you sitting there struggling with your food": experiences of adolescents and young adults with cerebral palsy.	The Netherlands	Qualitative	To find out which difficulties adolescents and young adults with cerebral palsy experience with eating and drinking in daily life and how they deal with these difficulties. Also, how they think about themselves with respect to eating and what does or does not help regarding social participation.	Total n= 10 CP= 10 Other= 0 Mean age: 18.5 years GMFCS: I-V	*Personal factors:* young adults' perception about eating and drinking in daily life.	adolescent
**Hanes et al. (2019) [77].** Beyond stereotypes of cerebral palsy: Exploring the lived experiences of young Canadians.	Canada	Qualitative	To blend lived experience with rigorous research methods to capture the intricate reality of young adults with CP—their physical, mental, and emotional health—as they navigate their life with a complex health condition.	Total n= 16 CP= 16 Other= 0 Mean age (CP): 26 years GMFCS: I-V	*Personal factors:* participants lived experiences in several áreas.	Adolescent /Young adult

**Lopes et al. (2019) [115].**<br><br>Social participation: the perspectives of adolescents with cerebral palsy and their mothers.	Brazil	Cross-sectional and exploratory study, with a qualitative design	To explore the perspectives of mothers and adolescents with CP on their social participation.	Total n= 14<br>CP= 7<br>Other= 7<br><br>Mean age (CP/Other): 13.8 /43 years<br><br>GMFCS: I-V	*Personal factors:* adolescents' and mothers perspectives about parenting and leisure/social participation.	Adolescent /Parent
**Bergqvist et al. (2019) [66].**<br><br>When I do, I become someone: experiences of occupational performance in young adults with cerebral palsy.	Sweden	Qualitative with aphenomenographic approach	To obtain deeper knowledge of how young adults with CP, having a relatively good motor function, perceive their occupational performance in everyday activities.	Total n= 10<br>CP= 10<br>Other= 0<br><br>Age range (CP): 19-30 years<br><br>GMFCS: I-III<br>MACS: I-II<br>CFCS: I-II	*Personal factors:* Open questions related to participants' experiences in occupational performance.	Young adult

**Grace et al. (2019) [116].**<br><br>Exploring participation experiences of youth who use AAC in social media settings: impact of an e-mentoring intervention.	Australia	Interventional	To examine the potential contributions of cross-age peer e-mentoring intervention (hereafter referred to as e-mentoring) to enhance participation in online conversations for young people who use augmentative and alternative communication (AAC).	Total n= 4<br>CP= 4<br>Other= 0<br><br>Mean age (CP): 16 years<br><br>GMFCS: I-IV<br>MACS: II-IV<br>CFCS: II-III	*Participation:* Self-Reported Experiences of Activity Settings.	Adolescent
**Capati et al. (2019) [46].**<br><br>Stander Use for an Adolescent with Cerebral Palsy at GMFCS Level with Hip and Knee Contractures.	The United States of America	Interventional	Can an adolescent with CP and 40-degree knee and/or hip flexion contractures benefit from a standing program, after being non-weight-bearing for many years?	Total n= 1<br>CP= 1<br>Other= 0<br><br>Mean age (CP): 16 years<br><br>GMFCS: V	*Activity:* Child Health Index of Life with Disabilities (CPChild Questionnaire).<br><br>*Body function:* postoperative pain.<br><br>*Body structure:* hip and knee range of motion (ROM).	Parent/ Clinician

**Power et al. (2020) [41].**<br><br>What predicts the proxy-reported health-related quality of life of adolescents with cerebral palsy in Bangladesh?<br>.	Australia	Exploratory study	To explore what factors predict the proxy-reported HRQoL of adolescents with CP in rural Bangladesh.	Total n= 154<br>CP= 154<br>Other= 0<br><br>Mean age (CP): 15.1 years<br><br>GMFCS: I-V	*Activity:* GMFCS.<br><br>*Participation:* Strengths and Difficulties Questionnaire (SDQ).<br><br>*Personal factors:* age, sex.<br><br>*Environmental factors:* school attendance and proxies of socioeconomic status such as monthly family income, household crowding, access to running water, and sanitation. Caregiver mental health.<br><br>*ICF not-covered:* Cerebral Palsy Quality of Life-Teens proxy report questionnaire (CPQoL-Teens).<br><br>*Body function:* type of CP, body mass index (BMI), other associated impairments.	Parent

**Jalón et al. (2020) [117].**<br><br>Did previous involvement in research affect recruitment of young people with cerebral palsy to a longitudinal study of transitional health care?	The United Kingdom	Not clearly specified.	To investigate whether being contacted about or participating in previous research and method of approaching potential participants (direct vs indirect) affected recruitment to a research study.	Total n= 410<br>CP= 410<br>Other= 0<br><br>Mean age (CP): 16.3 years<br><br>GMFCS: I-V	*Activity:* GMFCS<br><br>*Personal factors:* Date of birth, sex<br><br>*Environmental factors:* postcode<br><br>*ICF not-defined:* method and date of initial approach and decision about whether to join the transition study or not; number of previous studies young people had been approached about and the number they had joined.<br>———<br>*Body function:* CP subtype, intellectual ability	Data register

**van Gorp et al. (2020) [40].**<br><br>Childhood factors predict participation of young adults with cerebral palsy in domestic life and interpersonal relationships: a prospective cohort study.	The Netherlands	Longitudinal	To determine childhood predictors of participation in domestic life and interpersonal relationships of young adults with cerebral palsy (CP).	Total n= 67<br>CP= 67<br>Other= 0<br><br>Mean age: 24.6 years<br><br>GMFCS: I-V<br>MACS: I-V	*Activity:* VABS; GMFCS, MACS.<br><br>*Participation:* Life Habits Questionnaire (LIFE-H), VABS.<br><br>*Personal factors:* gender.<br><br>*Environmental factors:* housing, romantic relationship.<br><br>*Body function:* CP type.	Young adult
**Schmidt et al. (2020) [118].**<br><br>Autonomy in participation in cerebral palsy from childhood to adulthood.	The Netherlands	Longitidinal cross-sectional study	(1) describe the long-term development of autonomy in individuals with CP in several domains of participation from their teens into their early thirties and examine differences in development between individuals with high versus low levels of gross motor function; and (2) compare the proportions of individuals with CP who are autonomous in participation to the reference values of typically developing peers in the Netherlands.	Total n= 189<br>CP= 189<br>Other= 0<br><br>Mean age: 21.1 years<br><br>GMFCS: I-V	*Activity:* GMFCS<br><br>*Participation:* Rotterdam Transition Profile (RTP).<br><br>*Personal factors:* Age and level of education	Research records/ Young adult

**Jacobson et al. (2020) [73].**<br><br>Health-related quality of life, pain, and fatigue in young adults with cerebral palsy.	Sweden	Cross-sectional study	To describe health-related quality of life (HRQoL), pain, fatigue, and other health variables in young adults with cerebral palsy (CP), and to explore associations with the GMFCS and physical activity.	Total n= 61<br>CP= 61<br>Other= 0<br><br>Mean age: 21.2 years<br><br>GMFCS: I-V	*Activity:* level of physical activity<br><br>*Personal factors:* medical history and physical examination<br><br>*ICF not-covered:* health-related quality of life (HRQoL).<br><br>*Body function:* Brief Pain Inventory, Fatigue Severity Scale	Young adult
**Palisano et al. (2020) [17].**<br><br>Promoting capacities for future adult roles and healthy living using a lifecourse health developmen t approach.	The United States of America	Qualitative	To describe how young adults with CP experience lifecourse health development and create action-oriented messages for pediatric health service providers to promote children's capacities for future adult roles and healthy adult living	Total n= 23<br>CP= 23<br>Other= 0<br><br>Mean age (CP): 28.2 years<br><br>GMFCS: I-V<br>MACS: I-V<br>CFCS: I-V	*Activity:* GMFCS, MACS, CFCS.<br><br>*Personal factors:* gender; personal questions about perspectives on health living, education.<br><br>*Environmental factors:* residence, living situation, and employment.	Young adult

**Schwellnus et al. (2020) [58].**<br><br>A Solution-Focused Coaching Intervention with Children and Youth with Cerebral Palsy to Achieve Participation-Oriented Goals.	Canada	Multiple method approach	To investigate the impact of a Solution Focused Coaching intervention designed for pediatric rehabilitation (SFC-peds) on the attainment of participation goals for children/ youth with CP.	Total n= 12<br>CP= 12<br>Other= 0<br><br>Mean age (CP): 15 years<br><br>Functional classification not reported.	*Activity:* goal attainment scaling (GAS).<br><br>*Participation:* Canadian Occupational Performance Measure (COPM).<br><br>*Personal factors:* participants' experiences and satisfaction with the SFC-peds approach.	Adolescent /Parents
**Kwon et al. (2020) [119].**<br><br>Correlation between Accelerometer and Questionnaire-Based Assessment of Physical Activity in Patients with Cerebral Palsy.	Korea	Prospective	To investigate the correlation between accelerometer- and questionnaire-based assessment of PA in CP patients	Total n= 103<br>CP= 19<br>Other= 84<br><br>Mean age (CP): 19.7 years<br><br>Mean age (Other):29.2 years<br><br>GMFCS: I-III	*Activity:* accelerometer; International Physical Activity Questionnaire (IPAQ), Child Health Index of Life with Disabilities (CPChild Questionnaire).<br><br>*Participation:* Pediatric Outcomes Data Collection Instrument (PODCI).	Adolescent / Parent

**Walker et al. (2020) [120].**<br><br>Using participatory action research to examine barriers and facilitators to physical activity among rural adolescents with cerebral palsy.	The United States of America	Qualitative	To use a qualitative, community-based participatory action research method – Photovoice – to identify perceived facilitators and barriers to physical activity among adolescents with cerebral palsy (CP) in a rural community.	Total n= 15<br>CP= 7<br>Other= 8<br><br>Age range CP): 14-21 years<br><br>GMFCS: I-IV	*Activity:* Barriers to Physical Activity Questionnaire for People with Mobility Impairments (BPAQ-MI).<br><br>*Personal factors:* Photovoice: adolescent/parent perceptions/reflections about activity.	Adolescent /Parent

**Tu & Steinbok, (2020) [121].**<br><br>Long term outcome of Selective Dorsal Rhizotomy for the management of childhood spasticity-functional improvement and complications.	Canada	Review	To review the long-term outcomes in patients with cerebral palsy who underwent SDR in childhood.	Total n= 1.054<br>CP= diverse<br>Other= diverse<br><br>Mean age (CP): 16 years<br><br>GMFCS: I-V<br><br>MACS: I-V<br><br>CFCS/EDACS/VFCS: diverse	*ICF not-defined:* systematic database search.	Databases
**Jahnsen et al. (2020) [122].**<br><br>Independence of young people with cerebral palsy during transition to adulthood: a population-based 3 year follow-up study.	Norway	Cross-sectional/ longitudinal	To describe developmental and service transitions during a 3-year period among young people with cerebral palsy (CP)	Total n= 137<br>CP= 44<br>Other= 93<br><br>Mean age (CP/Other): 19.3/16.4 years<br><br>GMFCS: I-V<br>MACS: I-V	*Participation:* Rotterdam Transition Profile (RTP).	Adolescent

**Power et al. (2020) [123].**<br><br>'Flower of the body': menstrual experiences and needs of young adolescent women with cerebral palsy in Bangladesh, and their mothers providing menstrual support.	Australia	Qualitative	To describe the menstrual experiences and needs of young adolescent women with cerebral palsy (CP) in Bangladesh, and their mothers providing menstrual support.	Total n= 45<br>CP= 12<br>Other= 33<br><br>Mean age (CP/Other): 15 years / not reported<br><br>GMFCS: I-V	*Personal factors:* Participants' and parents' perspectives about menstruation.	Parents/ Adolescent

**Towns et al. (2020) [124].**<br><br>Balance confidence and physical activity participation of independently ambulatory youth with cerebral palsy: an exploration of youths' and parents' perspectives.	Canada	Qualitative descriptive methodology	1. To discover which physical activities youth with CP believe they do easily and those they avoid due to concerns about their balance; 2. To investigate youths’ perceptions of the influence of contextual factors on their capacity, performance, and participation; 3. To explore how youth with CP feel when they lose their balance. 4. To determine the extent to which the balance confidence of youth with CP influences their physical activity performance and participation.	Total n= 16<br>CP= 8<br>Other= 8<br><br>Mean age (CP/Other): 14 years / not reported<br><br>GMFCS: I-III	*Personal factors*: participants' and parents' perspectives about confidence and physical activity participation.	Adolescent /Parent
**Normann et al. (2020) [61].**<br><br>Navigating being a young adult with cerebral palsy: a qualitative study.	Denmark	Qualitative	To explore how living with CP affects young adults in general, and specifically which psychosocial, medical and healthcare needs are particularly important during this phase of life.	Total n= 6<br>CP= 6<br>Other= 0<br><br>Mean age (CP): 25.8 years<br><br>GMFCS: I-IV	*Personal factors:* Participants' perspectives about having CP and transitioning to adult life in various areas.	Young adult

**King et al. (2020) [76].**<br><br>Exploring the after-hours social experiences of youth with disabilities in residential immersive life skills programs: a photo elicitation study.	Canada	Interpretive phenomenological methodology	To provide a full picture of RILS programs, and gain a deeper understanding of why the “residential” aspect is important.	Total n= 5<br>CP= 5<br>Other= 0<br><br>Age range (CP): 16-21 years<br><br>Functional classification not reported.	*Personal factors:* Participants' perspectives about residential immersive life skills programs	Youth
**Pagliano et al. (2021) [42].**<br><br>Being adults with cerebral palsy: results of a multicenter Italian study on quality of life and participation.	Italy	Longitudinal multicenter study	To explore the quality of life and participation of adults with cerebral palsy.	Total n= 109<br><br>CP= 109<br>Other= 0<br><br>Mean age (CP): 26 years<br><br>GMFCS: I-V<br>MACS: I-V<br>CFCS: I-V	*Activity:* GMFCS, MACS, CFCS.<br><br>*Participation:* Life Habits Questionnaire (LIFE-H), aspects of daily life.<br><br>*Personal factors:* General state of health.<br><br>*Environmental factors:* Socio-economic status.<br><br>*ICF not-covered:* Short-Form health survey (SF-36).	Young adult

**Sentenac et al. (2021) [71].**<br><br>Disparity of child/parent -reported quality of life in cerebral palsy persists into adolescence.	France	Longitudinal	To examine child–parent discrepancy in reporting QoL in childhood and adolescence in a large European sample of children with CP and investigate the potential factors associated with such a discrepancy.	Total n= 354<br>CP= 354<br>Other= 0<br><br>Mean age (CP): 15.1 years<br><br>Functional classification not reported.	*Activity:* GMFCS.<br><br>*Personal factors:* Strengths and Difficulties Questionnaire (SDQ).<br><br>*Environmental factors:* child's school type; parents marital status; number of children at home; and highest educational qualification, Parenting stress.<br><br>*ICF not-covered*: Health Questionnaire for Children and Young People (KIDSCREEN-52).<br><br>*Body function:* pain; intellectual impairment; communication difficulties.	Adolescent /Parent
**Feitosa et al. (2021) [125].**<br><br>"Help Me to Improve my Own Priorities!": A Feasibility Study of an Individualized Intensive Goal Training for Adolescents with Cerebral Palsy.	Brazil	A single-group design with repeated measures	The primary outcome was the performance and satisfaction of the adolescents in prioritized goals. The secondary outcomes included adolescent's functional skills and independence in self-care, mobility and household tasks, participation at home, and manual dexterity. In addition, the preliminary effects of the individualized intensive training was documented.	Total n= 12<br><br>CP= 12<br>Other= 0<br><br>Mean age (CP): 15.2 years<br><br>GMFCS: I-IV<br>MACS: II-III	*Activity:* Pediatric Evaluation of Disability Inventory (PEDI), Box and Blocks Test<br><br>*Participation:* Canadian Occupational Performance Measure (COPM), Measure of Participation and Environment - Children and Youth (PEM-CY), Children's Questionnaire Helping: Responsibility, Expectation and Support (CHORES).<br><br>*Environmental factors:* participant's satisfaction with the intervention.<br><br>*ICF not-defined:* participant's adherence, adequacy of the instruments used	Parents/ Adolescent

**Shrader et al. (2021) [74].**<br><br>Well-Being of Ambulatory Adults With Cerebral Palsy: Education, Employment, and Physical Function of a Cohort Who Received Specialized Pediatric Care.	The United States of America	Prospective study	To assess independence, education, employment, walking activity, pain, utilization of health care, and physical function in an adult cohort of former patients from a pediatric CP specialty center.	Total n= 126<br><br>CP= 126<br>Other= 0<br><br>Mean age (CP): 29.7 years<br><br>GMFCS: I-III	*Activity:* community walking activity, Independence<br><br>*Personal factors:* sex, age, ethnicity, race, etc; Satisfaction with Life Scale<br><br>*Environmental factors:* education, employment, income, health care utilization.<br><br>*Body function:* physical function, pain, and pain interference.	young adult

**Bagazgoïtia et al. (2021) [11].**<br><br>Quality of Life in Young Adults With Cerebral Palsy: A Longitudinal Analysis of the SPARCLE Study.	France	Longitudinal	To identify how the QoL of individuals with CP evolves from childhood to young adulthood and whether the severity of impairment and frequency of pain and seizures affect their QoL. We also aimed to determine whether different shapes of QoL trajectories exist in order to better understand the impact of impairment and comorbidities on QoL.	Total n= 164<br><br>CP= 164<br>Other= 0<br><br>Mean age (CP): 24.3 years<br><br>GMFCS: I-V	*Activity:* GMFCS, Bimanual Fine Motor Function (BFMF).<br><br>*ICF not-covered:* Health Questionnaire for Children and Young People (KIDSCREEN), World Health Organization Quality of Life Brief Version (WHOQOL-Bref).<br>———<br>*Body function:* frequency of pain, intellectual ability, subtype of CP.	Young adult
**Lennon et al. (2021) [48].**<br><br>Mobility and gait in adults with cerebral palsy: Evaluating change from adolescence.	The United States of America	Longitudinal	1) Evaluate changes in gait and mobility and examine factors that influence change from adolescence to adulthood in people with CP, and 2) Describe self-reports of pain, participation, depression, and physical function of adults with CP.	Total n= 136<br><br>CP= 136<br>Other= 0<br><br>Age range (CP): 16-29 years<br><br>GMFCS: I-IV	*Activity:* gait kinematic, Functional Mobility Scale (FMS), Gross motor function measure (GMFM).<br><br>*Participation:* Patient-Reported Outcomes Measurement Information System (PROMIS-participation).<br>———<br>*Body function:* Ashworth Scale; physical function, pain interference, Patient-Reported Outcomes Measurement Information System (PROMIS-depression).	Adolescent

**Lai et al. (2021) [126].**<br><br>Leisure-time physical activity interventions for children and adults with cerebral palsy: a scoping review.	The United States of America	Scoping review	(1) to identify and report response rates of children and adults with CP to key stages of leisure-time physical activity (LTPA) interventions (i.e. recruitment, screening and enrollment, intervention, and follow-up); (2) to describe intervention effects by age group (children and adults) and functional mobility level (Gross Motor Functional Classification System [GMFCS] level); and (3) to evaluate the quality of the published RCTs of LTPA.	Total n= 1.513<br>CP= diverse<br>Other= diverse<br><br>Mean age (CP): 13 years<br><br>GMFCS: I-V<br><br>Functional classification: diverse	*ICF not-defined:* systematic database search.	Databases
**Kusumoto et al. (2021) [49].**<br><br>Relevant factors of self-care in children and adolescents with spastic cerebral palsy.	Japan	Cross-sectional	To investigate the factors that influence self-care capability and performance in children and adolescents with spastic CP.	Total n= 76<br><br>CP= 76<br>Other= 0<br><br>Mean age (CP): 13.6 years<br><br>GMFCS: I-IV<br>MACS: I-V	*Activity:* Pediatric Evaluation of Disability Inventory (PEDI), Box and Blocks test; MACS, GMFCS.	Adolescent

**Dehghanizadeh et al. (2021) [62].**<br><br>Exploring the contributing factors that influence the volition of adolescents with cerebral palsy: A directed content analysis.	Iran	Qualitative	To explore the elements contributing to the volition of adolescents with CP based on their own experience.	Total n= 10<br>CP= 5<br>Other= 5<br><br>Mean age (CP/O): 14.6 / 43.6 years<br><br>GMFCS: I-III	*Personal factors:* Personal perspectives about volition	Adolescent /Parent
**Gray et al. (2021) [78].**<br><br>Puberty and menarche in young females with cerebral palsy and intellectual disability: a qualitative study of caregivers' experiences.	USA	Phenomenological qualitative study	To explore the experiences of parents of young females with cerebral palsy (CP) and intellectual disability at the onset of puberty.	Total n= 9<br>CP= 0<br>Other= 9<br><br>Age range (CP):<br>14-24<br><br>GMFCS: IV-V	*Personal factors:* Parents' perspectives about puberty.	Parent

Legend: GMFCS: Gross Motor Function Classification System; MACS: Manual Ability Classification System; CFCS: Communication Function Classification System; EDACS: Eating and Drinking Ability Classification System; VFCS: Visual Function Classification System.
