# Supplementary material for: Looking beyond Body Structure and Function: ICF Foci and Who Is Being Assessed in Research about Adolescents and Young Adults with Cerebral Palsy—A Scoping Review
Source: Int J Environ Res Public Health. 2024 May 24;21(6):670. doi: 10.3390/ijerph21060670 (PMC11203594; doi:10.3390/ijerph21060670)
Supplement: Supplementary file 1 [file ijerph-21-00670-s001.zip › S2 Complete search strategy EMBASE.pdf]

S2 Appendix 1. Complete search strategy EMBASE example.

**Looking beyond Body Structure and Function: ICF Foci and Who Is Being Assessed in Research about Adolescents and Young Adults with Cerebral Palsy— A Scoping Review (Santana et al., 2024)**

**Full search string used at Embase database:**

Fixed concepts

**Cerebral Palsy:** cerebral palsy/; cerebral palsy.mp.

AND

**Youth:** adolescent/; youth.mp.; adolescen*.mp.; young adult/; young adult.mp.

AND

Variable concepts

**Health development:** health care access.mp.; health care delivery.mp.; health service/; treatment delivery.mp.; health attitude.mp.; attitude to health/; patient compliance/; health knowledge.mp.

OR

**Participation:** community participation/; stakeholder engagement/; participat*.mp.; social participation/; patient participation/.

OR

**Independence:** self care/; independent living/; social support/; social environment/; daily life activity/; autonom*.mp.; personal autonomy/; independence/.
